# Effect of Co-Composting Cattle Manure with Construction and Demolition Waste on the Archaeal, Bacterial, and Fungal Microbiota, and on Antimicrobial Resistance Determinants

**DOI:** 10.1371/journal.pone.0157539

**Published:** 2016-06-14

**Authors:** Devin B. Holman, Xiying Hao, Edward Topp, Hee Eun Yang, Trevor W. Alexander

**Affiliations:** 1 Lethbridge Research Centre, Agriculture and Agri-Food Canada, Lethbridge, AB, Canada; 2 Agriculture and Agri-Food Canada, Southern Crop Protection and Food Research Centre, London, ON, Canada; Purdue University, UNITED STATES

## Abstract

Agricultural operations generate large quantities of manure which must be eliminated in a manner that is consistent with public health guidelines. Meanwhile, construction and demolition waste makes up about 25% of total solid municipal waste. Co-composting of manure with construction and demolition waste offers a potential means to make manure safe for soil amendment and also divert construction and demolition waste from municipal landfills. Therefore, the archaeal, bacterial, and fungal microbiota of two different types of composted cattle manure and one co-composted with construction and demolition waste, were assessed over a 99-day composting period. The microbiota of the three compost mixtures did not differ, but significant changes over time and by sampling depth were observed. *Bacillus* and *Halocella*, however, were more relatively abundant in composted manure from cattle fed dried distillers’ grains and solubles. *Proteobacteria* and *Bacteroidetes* were enriched at day 0 and *Firmicutes* at day 99. The fungal genus *Kernia* was the most relatively abundant overall and was enriched at day 0. The concentration of 12 antimicrobial resistance determinants in the compost mixtures was also determined, and 10 of these determinants decreased significantly from days 0 to 99. The addition of construction and demolition waste did not affect the persistence of antimicrobial resistance genes or community structure of the compost microbiota and therefore co-composting construction and demolition waste with cattle manure offers a safe, viable way to divert this waste from landfills.

## Introduction

Agricultural operations often produce large amounts of manure which if applied directly to agricultural land can have a negative effect on soil, water, and air quality through contamination, odour and gas emissions, and nutrient leaching [1]. Manure is also a reservoir of antimicrobial-resistant bacteria and the levels of resistant bacteria and resistance determinants in feces can increase following administration of antimicrobials to livestock [2–4]. However, even when livestock are not administered antimicrobials, a background level of resistance exists in feces which can fluctuate based on feed ingredients in animal diets [5–7]. If left unattended, the levels of resistant bacteria in feces or manure can increase over time, resulting in greater concentrations compared to freshly deposited material [8].

The composting of manure results in a product that is more nutrient-stable and free of microbial pathogens and phytotoxins [9]. Furthermore, composting may also decrease the concentration of excreted antimicrobials, resistance determinants, and resistant bacteria [10–12]. Transportation costs are also reduced as composting significantly decreases the volume and mass of the manure [13]. Composting is a largely aerobic and natural process involving the biodegradation of organic matter and is often described as having three microbiological phases: 1) an initial mesophilic phase (20 to 40°C) from 1 to 3 days, 2) a thermophilic phase (35 to 65°C), and 3) a cooling phase where mesophiles increase in abundance again as the temperature falls [14,15]. There is also a last stage in the composting process termed the maturation or curing phase, where microbial activity is reduced. This phase is necessary to ensure that phytotoxins and environmentally harmful products have been removed from the compost [14,16].

Although bacteria and fungi are both important contributors to the composting process, bacteria are more abundant and are largely responsible for heat generation and the degradation of compost substrates [17]. Heat, along with carbon dioxide (CO_2_), ammonia (NH_3_), water, and organic acids, is produced during the metabolism of organic matter during the mesophilic stage. As a result of this subsequent increase in temperature, thermophilic bacteria thrive and become dominant at this stage [14]. This thermophilic phase is particularly important from an agricultural perspective as the elevated temperature is responsible for eliminating pathogens and weed seeds [17].

In Alberta, Canada, approximately 25% of total municipal solid waste is comprised of construction and demolition (C&D) waste [18]. Within North America, C&D waste is made up of around 20 to 30% wood and 5 to 15% drywall [18]. Two of the strategies for diverting C&D waste from landfills are to use the wood and drywall components of C&D waste as a bedding material for feedlot cattle in place of straw and to use these fractions as a bulking agent in composting manure [18,19]. Previously it was demonstrated that the addition of C&D waste to feedlot cattle manure prior to composting resulted in composted manure with a higher sustained temperature, lower water content, higher pH, and altered mineral contents [18]. However, the effect that these changes have on the microbial community during the composting process is also an important consideration, given that composting is generally considered a method to reduce antimicrobial-resistant and pathogenic bacteria in livestock feces. Therefore, the objective of the current study was to investigate the dynamics of the archaeal, bacterial, and fungi microbiota in two different types of composted feedlot cattle manure and to determine what effect the addition of C&D waste has on these microbiota, over a 99-day period. The effect that these compost mixtures have on the concentration of antimicrobial resistance determinants in the cattle manure was also assessed. It was hypothesized that the addition of C&D waste would not affect the microbial parameters of composting.

## Materials and Methods

### Experimental design and sampling

Detailed descriptions of the experimental design can be found in Hao et al. [18]. Briefly, manure from feedlot cattle fed either a barley-based control diet (CON) or corn dried distillers grain and solubles (DDG) diet was composted over a 99-day period. The diet fed to CON cattle contained 860 g rolled barley grain, 100 g barley silage, and 40 g supplement kg^−1^ dry matter [18]. The DDG diet was similar to the CON diet but with 300 g kg^-1^ corn dried distillers’ grains with solubles replacing an equal amount of barley grain. A complete description of the chemical composition of the CON and DDG manure was reported previously [18]. Compared to manure from cattle fed the CON diet, manure from cattle on the DDG diet had higher δ15N, water-soluble N, and water-extractable NH_4_^+^, PO4^3−^, SO_4_^2−^, and Mg contents. The two manures had similar pH, electrical conductivity, total carbon, non-purgeable water soluble organic carbon, total nitrogen, δ13C, C/N ratio, total phosphorous, water-extractable K and Ca, and volatile fatty acid content. Manure from cattle on the barley-based diet was also amended with construction and demolition waste (CON C&D) in a 4:1 ratio (weight:weight basis) on day 0 of the study. The manure was sourced from cattle that did not receive any antimicrobial agents. The construction and demolition waste was mostly wood and drywall (gypsum). Compost starting material was placed in bins that were made from rectangular-shaped cereal straw bales with a volume of 13 m^3^ (2.5 m long x 2.22 m wide x 2.35 m high). Each compost mixture type was replicated three times and turned on days 14, 37, and 64 of the composting experiment. This experiment was performed at the Lethbridge Research and Development Centre (Lethbridge, Alberta, Canada) with permission from the Proposal Review Committee and Associate Director of Research, Development and Technology.

Samples were taken from each compost bin on days 0 (mesophilic phase), 14 (prior to turning; thermophilic phase), and 99 (cooling/maturation phase) of the experiment and were obtained from both the top (7 cm below the surface) and middle (≈ 90 cm below the surface) depths. Samples were taken only at the top of the compost pile on day 0 as the compost pile was homogenous at this point. Two depths were chosen for analysis as it has previously been shown that bacterial communities vary according to compost pile depth [20]. At all sampling time points, triplicate subsamples (approximately 200 g) were randomly collected from respective depths and mixed together into a single pooled sample. Samples on days 14 and 99 were collected after a front-end loader removed approximately half of the compost pile, exposing the complete pile from top to bottom. At that point, depths were measured and the 7-cm and 90-cm subsamples were collected. Pooled samples were stored at -80°C for DNA analysis. For the analysis of fungal diversity, only samples from the middle depth in the CON and CON C&D compost bins were used.

### DNA extraction

Total genomic DNA was extracted using the PowerLyzer PowerSoil DNA isolation kit (MoBio, Carlsbad, CA, USA) according to manufacturer’s instructions with the following modifications: samples were lyophilized prior to DNA extraction and only 100 mg of dried compost was used for extraction, samples were incubated at 65°C for 10 min prior to bead-beating, and the beat-beating step was performed using a TissueLyzer LT (Qiagen Canada Inc., Toronto, ON, Canada) for 1 min at 30 Hz.

### Amplification and sequencing of the archaeal and bacterial 16S rRNA gene

The primers 515-F (5'-GTGCCAGCMGCCGCGGTAA-3') and 806-R (5'-GGACTACVSGGGTATCTAAT-3') were used to amplify the V4 region of Archaea and Bacteria [21]. PCR amplification and sequencing was performed at Molecular Research LP (Shallowater, TX). Briefly, the 16S amplicons were generated using HotStarTaq Plus Master Mix Kit (Qiagen, Valencia, CA). The PCR program consisted of a 3 min initial denaturation at 94°C followed by 28 cycles of 94°C for 30 s, 53°C for 40 s, and 72°C for 1 min, with a final extension of at 72°C for 5 min. The size and specificity of PCR amplicons were verified using 2% agarose gel electrophoresis and samples were then pooled together in equal proportions and prepared for sequencing according to the standard protocol for the Illumina TruSeq DNA library preparation kit (Illumina, San Diego, CA). Sequencing was carried out using an Illumina MiSeq system (2 x 300 bp) following manufacturer’s instructions.

### Amplification and sequencing of the fungal ITS region

The fungal internal transcribed spacer 1 (ITS1) and ITS2 regions were amplified using the primers ITS1-F (5'-CTTGGTCATTTAGAGGAAGTAA-3') [22] and ITS4-R (5'-TCCTCCGCTTATTGATATGC-3') [23] together with the same PCR conditions as for the 16S rRNA gene. The 16S rRNA gene and ITS primer pairs contained 8-bp barcodes that were unique for each sample.

### Sequence analysis

Both 16S rRNA gene and ITS sequences were processed using the QIIME software package (version 1.9.1) [24]. For archaeal and bacterial 16S rRNA gene sequences, the forward reads were used for analysis as the amplicon size was 291 bp in total and the forward read length was 300 bp. All samples were demultiplexed and quality filtered with the removal of primers, barcodes, and sequences with an average Phred score of at least 25. The UCHIME algorithm [25] implemented in USEARCH (version 6.1544) [26] was then used to remove chimeric sequences. The remaining high quality 16S rRNA gene sequences were clustered into OTUs (operational taxonomic units) at 97% similarity using the open-reference OTU picking method and the SILVA database (version 111) [27]. Sequences that did not match OTUs in the SILVA database were then clustered into OTUs using the de novo approach and USEARCH (version 6.1544). Taxonomy was assigned using UCLUST consensus taxonomy assigner [26] and the SILVA database with a minimum similarity of 0.9 and max accepts of 3. PyNAST [28] was used to align the representative sequences for each OTU and a phylogenetic tree was created using FastTree [29].

For fungal ITS sequences, the forward and reverse fungal ITS reads were joined using Seqprep (https://github.com/jstjohn/SeqPrep), quality filtered as above, and chimeras were also removed using USEARCH. Non-chimeric ITS sequences were then clustered into OTUs at 97% similarity using the open reference OTU picking method and the fungal UNITE ITS 12_11 database [30]. Taxonomy was assigned using the BLAST method against the UNITE database. OTUs containing less than 10 sequences were removed from the 16S rRNA gene and fungal ITS data sets prior to downstream analysis. In addition, all samples were randomly subsampled and rarefied at 22000 sequences and 3100 for 16S rRNA gene and fungal ITS data sets respectively, to ensure that each sample had an equal number of sequences for analysis. The representative sequences from the 50 most relatively abundant OTUs that were unassigned taxonomy at the phylum-level were used to classify these OTUs using BLASTn (https://blast.ncbi.nlm.nih.gov/Blast.cgi; accessed February 9, 2016).

### Statistical analysis

Archaeal, bacterial, and fungal diversity in each sample was calculated within QIIME using the Chao1 [31] and Shannon index [32]. The phylogenetic diversity (PD whole tree) [33] was also calculated for the archaeal and bacterial data set. Diversity metrics were analyzed using the PROC MIXED procedure in SAS 9.4 (SAS Inst., Inc., Cary, NC, USA) with sampling time as a repeated measure. Manure type (M), sampling depth (D), and sampling time (T in wk), M x D, M x T, and M x D x T were the fixed effects and replicate x manure type was included as the random effect. Results were considered statistically significant at P < 0.05. The bacterial and archaeal community structure (beta diversity) of each manure type, sampling time, and sampling depth was evaluated using the weighted UniFrac distances [34] and visualized as principal coordinate analysis (PCoA) plots using Emperor [35]. The fungal community structure was assessed using Bray-Curtis distances and PCoA plots.

Linear discriminant analysis effect size (LEfSe) was used to determine which phyla and genera were significantly different based on manure type, sampling time, and sampling depth. Significantly different (P < 0.05) taxa among groups of samples are identified by LEfSe using the Kruskal-Wallis test and the effect size of each of these is estimated using linear discriminant analysis [36]. A LDA score of 3.0 was used as the cut-off for plotting differentially abundant genera. ANOSIM (analysis of similarities) with 999 permutations was used to compare the weighted UniFrac and the Bray-Curtis distances.

Sequences were submitted to the NCBI Sequence Read Archive (http://www.ncbi.nlm.nih.gov/sra) under BioProject accession PRJNA309707.

### Quantitative PCR

Macrolide (*erm*(A), *erm*(B), *erm*(F), *erm*(X)), sulphonamide (*sul*(1), sul(2)), and tetracycline (*tet*(B), *tet*(C), *tet*(H), *tet*(L), *tet*(M), *tet*(W)) resistance determinants were quantified using quantitative PCR (qPCR). These resistance determinants were chosen based on their widespread dissemination and prevalence in the manure of feedlot cattle [37]. Primers and annealing temperatures used for qPCR assays are listed in S1 Table.

All qPCR reactions were conducted on a System C1000 Touch Thermal Cycler with a CFX96 Real Time system (Bio-Rad Laboratories, Mississauga, ON, Canada) and each reaction contained: 1x iQ SYBR Green Supermix (Bio-Rad Laboratories), 20 ng of DNA, 0.3 μM of each primer, 88 ng μl^-1^ bovine serum albumin (New England Biolabs, Pickering, ON, Canada), and sterile, nuclease-free water in a total volume of 25 μl. Primer concentrations were increased to 0.4 μM for *tet*(H) and 0.6 μM for *erm*(A). The qPCR conditions consisted of an initial denaturation at 95°C for 3 min, followed by 40 cycles of 15 sec at 95°C, 1 min at the annealing temperature, and 1 min at 72°C. To ensure only a single product was being amplified, melt curve analysis was performed at the end of each qPCR run using a temperature range of 65°C to 95°C with fluorescence measured at 0.5°C intervals. Bio-Rad CFX Manager 3.1 (Bio-Rad Laboratories) was used for data analysis and all qPCR reactions were carried out in triplicate.

Standards for qPCR were prepared as previously described in Alexander et al. [37], using a TOPO TA cloning kit (Life Technologies, Burlington ON, Canada), and an One Shot TOP10 competent cells (Life technologies). The concentration of each resistance determinant (copies g^-1^ compost dry weight) was analyzed using the PROC MIXED procedure as described above. The PROC CORR procedure was used in SAS 9.4 (SAS Inst.) to calculate the Pearson correlation coefficients between the concentration of each resistance determinant and the relative abundance of the 20 most prevalent bacterial genera.

## Results and Discussion

### Archaeal and bacterial 16S rRNA gene sequencing results

A total of 2,362,054 quality-filtered and chimera-checked 16S rRNA gene sequences were obtained with an average length of 270 bp. There were 14,656 unique archaeal and bacterial OTUs identified across all compost samples, although the 100 most relatively abundant OTUs accounted for 41.6% of the total sequences (data not shown). Overall, 99% of the sequences were classified at the phylum level. While 29 different phyla were identified across all compost samples, more than 85% of total sequences were classified as *Firmicutes*, *Actinobacteria*, or *Proteobacteria* (S2 Table). Archaeal sequences comprised 1.2% of total 16S rRNA gene sequences with only two phyla identified: *Euryarchaeota* and *Thaumarchaeota*. Of these two archaeal phyla, *Euryarchaeota* was over 100 times as relatively abundant.

**Fig 1:**
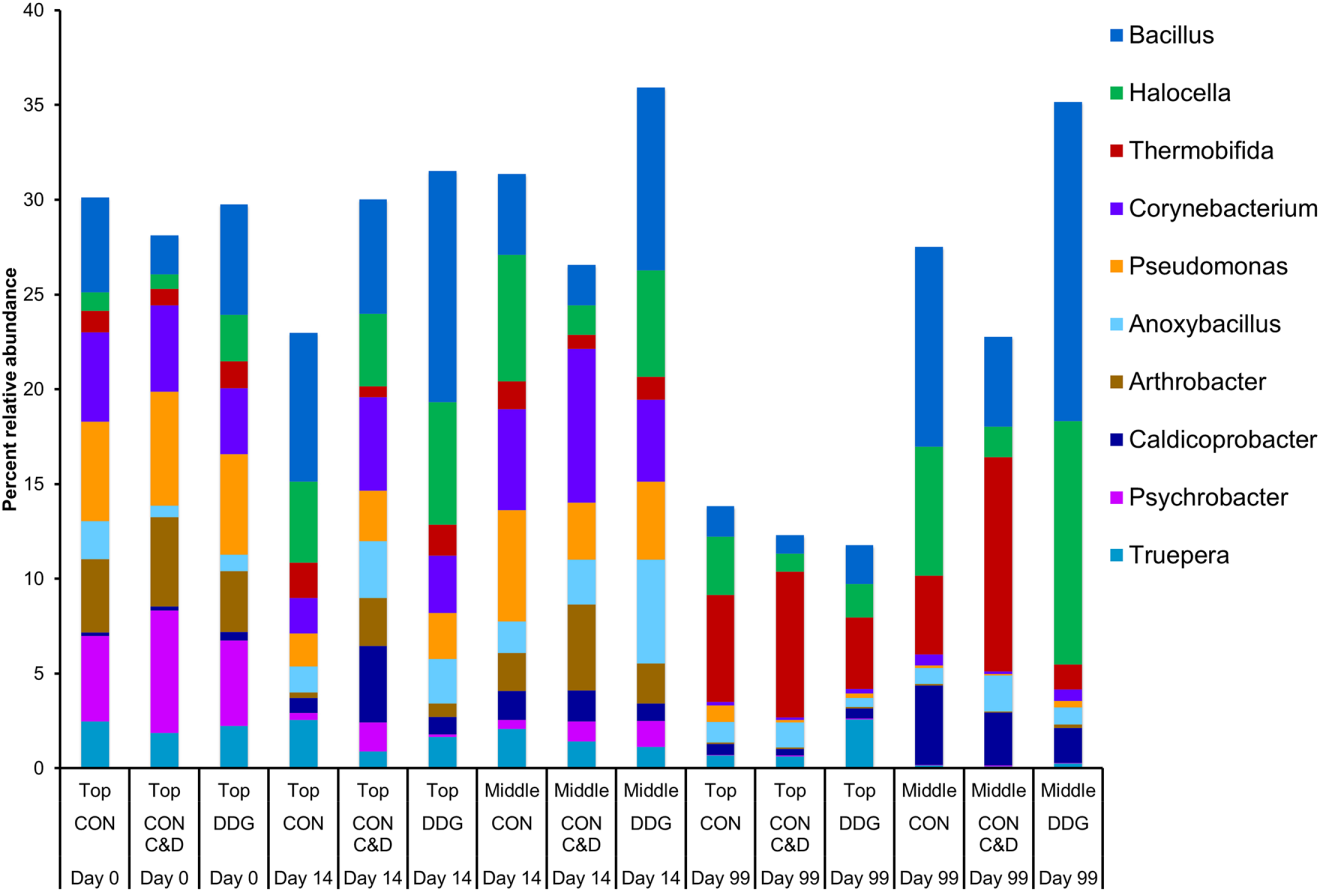
The 10 most relatively abundant bacterial genera in the each compost mixture type at each sampling time and sampling depth (n = 3).

The 16S rRNA gene sequences were classified into 422 different archaeal and bacterial genera, representing 58.8% of the total sequences. Only eight of these genera belonged to the *Archaea* domain, with *Methanobrevibacter* and *Methanothermobacter* being the only two with a relative abundance greater than 0.1%. Overall, *Bacillus*, *Halocella*, and *Thermobifida* were the most relatively abundant bacterial genera. The ten most relatively abundant genera overall are displayed by sampling time, sampling depth, and compost mixture type in Fig 1. The number of OTUs in each sample ranged from 1445 to 3009 while the Shannon index was between 4.00 and 6.51 for all samples (Table 1). The core microbiota of the composting process, defined as those OTUs found in all compost sample types, depths, and sampling times, was comprised of 38 OTUs and the large majority of these OTUs (74%) belonged to the *Firmicutes* phylum (S3 Table).

**Table 1:** 16S rRNA gene sequence richness and diversity of composted cattle manure by time and sampling depth.

Treatment^a^	Time (day)	Depth^b^	Number of OTUs	Shannon	Chao1	PD Whole Tree
CON	0	Top	2808 ±185a	6.31 ±0.08	4699 ±369a	185.7 ±2.2a
DDG	0	Top	2544 ±208ab	6.13 ±0.33	4483 ±432abc	180.2 ±14.3a
CON C&D	0	Top	2645 ±176ab	6.27 ±0.08	4505 ±473ab	184.9 ±7.5a
CON	14	Top	2471 ±282ab	6.08 ±0.47	4351 ±376abc	174.4 ±18.9ab
CON	14	Middle	2757 ±58a	6.25 ±0.02	4844 ±134a	181.9 ±0.9a
DDG	14	Top	2473 ±140ab	5.88 ±0.32	4619 ±237a	174.6 ±12.1ab
DDG	14	Middle	2455 ±456abc	5.93 ±0.76	4468 ±658abc	169.4 ±22.8ab
CON C&D	14	Top	2150 ±219abc	5.88 ±0.36	3844 ±126abcd	158.1 ±17.1ab
CON C&D	14	Middle	2594 ±262ab	6.04 ±0.28	4643 ±473ab	183.6 ±13.6a
CON	99	Top	1981 ±201abc	5.82 ±0.43	3486 ±63abcd	156 ±16.1ab
CON	99	Middle	1768 ±148bc	5.09 ±0.09	3468 ±152abcd	137.4 ±11.8ab
DDG	99	Top	1788 ±143bc	5.74 ±0.16	3010 ±240abcd	148.3 ±9.3ab
DDG	99	Middle	1750 ±327bc	4.77 ±0.77	3399 ±521cd	139.2 ±17.8ab
CON C&D	99	Top	1689 ±169bc	5.48 ±0.29	3139 ±208bcd	136.5 ±10.3ab
CON C&D	99	Middle	1538 ±12c	5.08 ±0.01	2792 ±137d	119.8 ±4.6b

^a^ CON, manure from cattle on a control diet of barley; DDG, manure from cattle fed dried distillers grains and solubles; CON C&D, manure from the control diet cattle amended with construction and demolition waste. Mean ± standard deviation (n = 3) is shown for compost. Means with different lowercase letters are significantly different from one another (P<0.05).

^b^ Samples were taken at the top (7 cm) and middle (90 cm) of each compost bin.

### Effect of compost mixture type on the archaeal and bacterial compost microbiota

**Fig 2:**
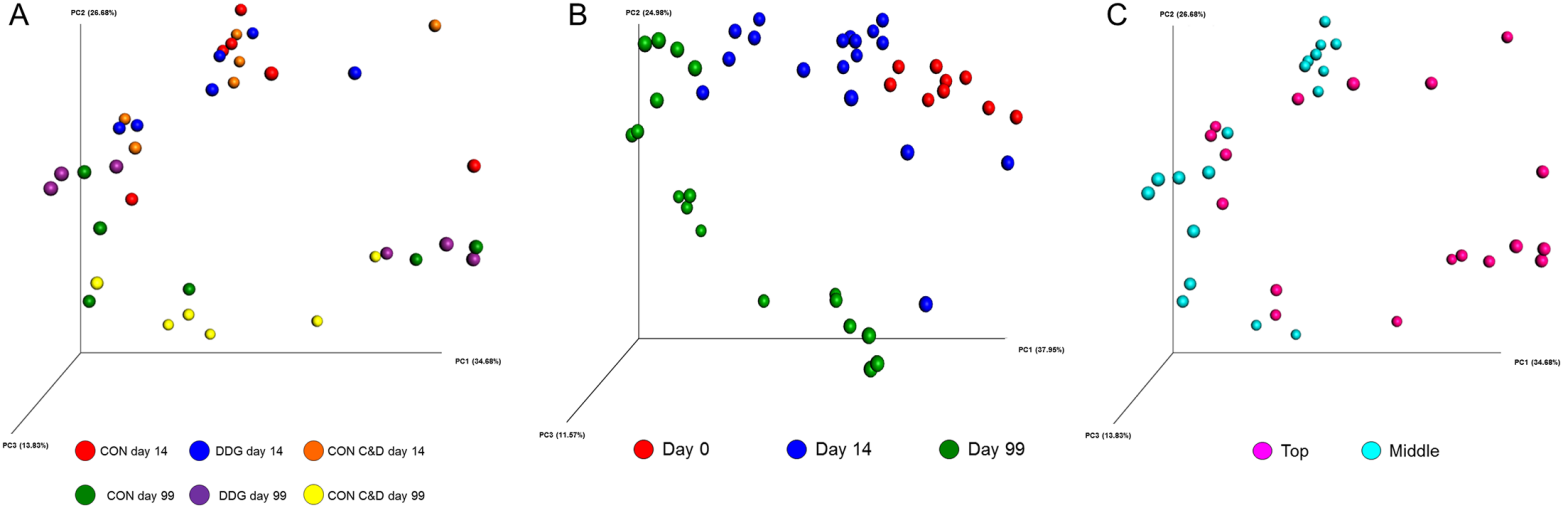
Principle coordinate analysis (PCoA) plots of the weighted UniFrac distances for the archaeal and bacterial microbiota of each A) compost mixture type x sampling time, B) sampling time, and C) sampling depth (day 0 sample excluded). CON = composted manure from cattle on a control diet of barley, DDG = composted manure from cattle fed dried distillers grains and solubles, and CON C&D = composted manure from the control diet cattle amended with construction and demolition waste.

The archaeal and bacterial microbiota was compared among the three different compost mixture types (CON, DDG, and CON C&D). Despite the fact that the CON C&D compost had a lower pH, water, and volatile fatty acid content, as well as a higher C:N ratio and a higher sustained temperature [18], these differences did not result in an archaeal and bacterial microbiota that was significantly altered from the CON and DDG compost mixtures. Compost mixture type had no effect on any of the archaeal and bacterial diversity and richness measures at either day 14 or 99 (Table 1; P > 0.05). In addition, when the structure of the archaeal and bacterial communities was assessed using weighted UniFrac distances, compost mixture type also had no significant impact on archaeal and bacterial community structure (P > 0.05) as seen in the PCoA plots of sampling time x compost mixture type (Fig 2A).

**Fig 3:**
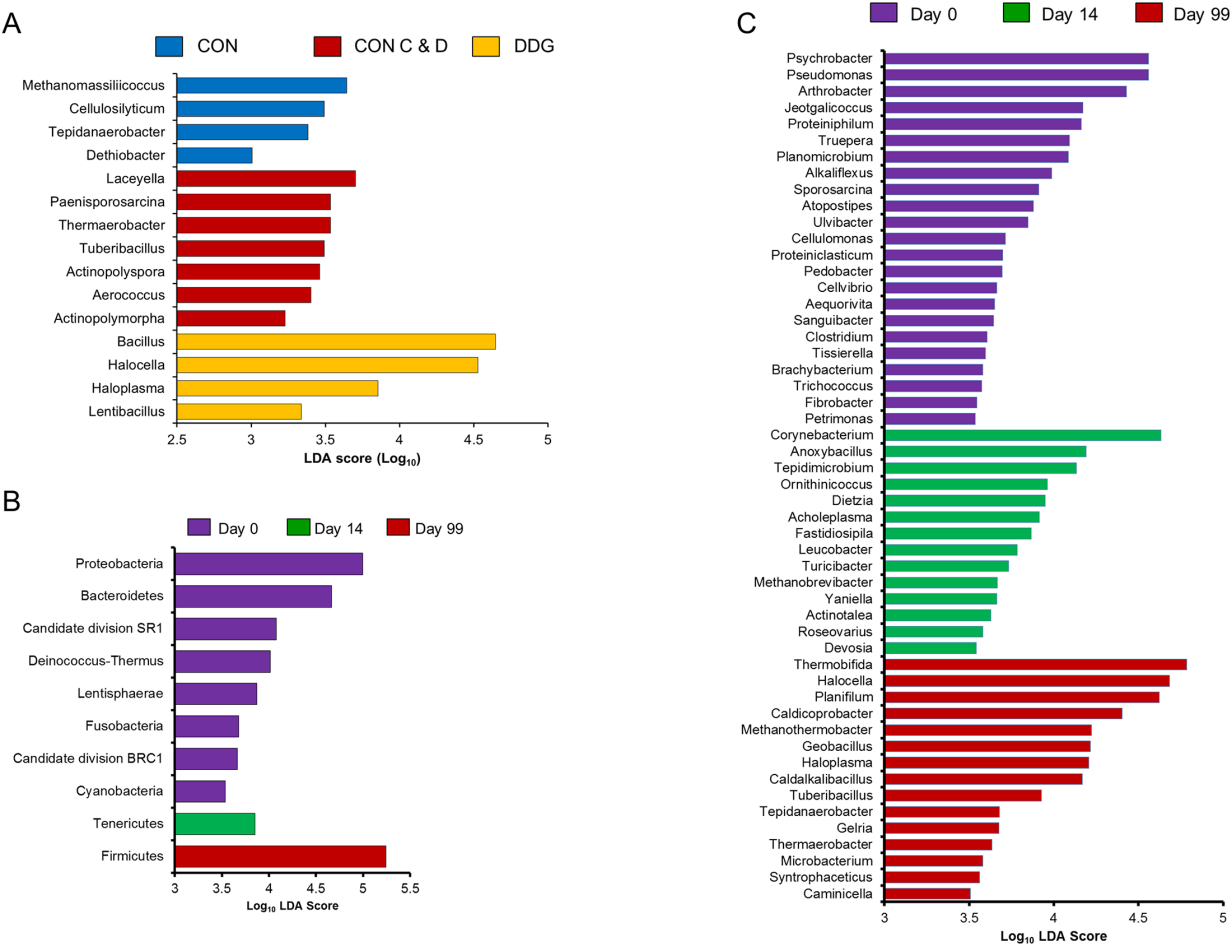
Differentially abundant archaeal and bacterial taxa as assessed using linear discriminant analysis (LDA) with effect size measurements (LEfSe). In A) genera enriched in one of the three compost mixture types, B) phyla, and C) genera enriched at one of the three sampling times. For A) only genera with a LDA score greater than 3.0 are displayed, and in B) and C) only those taxa with a LDA score greater than 3.5. CON = composted manure from cattle on a control diet of barley, DDG = composted manure from cattle fed dried distillers grains and solubles, and CON C&D = composted manure from the control diet cattle amended with construction and demolition waste.

Linear discriminant analysis effect size (LEfSe) was used to determine which phyla and genera were enriched among the three compost mixture types. There were 15 genera identified that were more relatively abundant in one of the compost types (Fig 3A; LDA score greater than 3.0). Most notably, *Bacillus* and *Halocella* were more enriched in the DDG compost (LDA score greater than 4.5). *Bacillus* spp. are often reported to be dominant in the thermophilic phase of the composting process [38] likely due to the increased heat-tolerance of some members of this genus [39], although it is not clear why this genus might be associated with this type of compost. The *Halocella* genus, which was most relatively abundant in the middle sampling depth of the DDG compost, has only one species, *Halocella cellulosilytica*, a moderately halophilic, anaerobic cellulolytic bacterium [40]. The DDG compost did have significantly higher concentrations of ammonium (NH_4_^+^), Mg^2+^, and total phosphorus, compared to the CON and CON C&D and inclusion of distillers’ grains in cattle diets typically increases the overall fibre content [11]. Thus, the increase in *Halocella* observed in the DDG compost may have resulted from differences in chemical composition compared to CON substrates, particularly the higher fibre content of the DDG treatment. There were four different genera that were more relatively abundant in the CON compost, however, only two, *Tepidanaerobacter* and *Dethiobacter*, were identified in all CON samples, at a relative abundance of 0.52 and 0.14%, respectively. While it is not clear what factors in the CON compost may have contributed to their increased relative abundance, members of the *Tepidanaerobacter* and *Dethiobacter* genera belong to the Clostridia class and are anaerobic and thermotolerant rods that have only recently been described [41,42].

While seven genera were found to be enriched in the CON C&D compost, all of these were relatively rare genera with an overall relative abundance of less than 0.01%, although four of these genera are known to contain thermophilic species, *Actinopolyspora*, *Laceyella*, *Thermoanaerobacter*, and *Tuberibacillus*. None of these genera were common indicator bacteria (e.g. *Enterococcus*) that are associated with fecal contamination or are potential zoonotic pathogens. Given the higher sustained temperature and altered chemical properties of the C&D compost [18], it is not immediately clear why the addition of C&D waste to the CON manure did not result in an archaeal or bacterial microbiota that was significantly different from the other two compost mixtures. However, several factors may explain this finding. One potential reason is that the starting material (i.e., cattle manure) was largely the same for all three compost mixtures and therefore a similar microbiota was already present on day 0. Furthermore, all of the starting manure was composted in the same manner for all three mixture types and for the same length of time. Although the temperature remained above 55°C in the CON C&D compost for a longer period of time, none of the compost mixtures had reached mesophilic temperatures again by the end of the experiment. Therefore, the high temperatures observed throughout the study would have exerted a strong selective pressure that may have changed the structure of the microbiota of all compost mixture types in a similar way. In further support of this hypothesis, a recent study by de Gannes et al. [43] also noted that the archaeal and bacterial community structure was not altered during composting by either the addition of coffee hulls, rice straw, or sugarcane bagasse to cattle or sheep manure.

### Effect of sampling depth and time on the archaeal and bacterial compost microbiota

Sampling time had the greatest impact on the archaeal and bacterial microbiota, as all compost mixture types had progressively and significantly lower archaeal and bacterial diversity and richness from day 0 to 99 (Table 1; P < 0.05). A total of 10 phyla and 52 genera were enriched at one of the three sampling times, including several that were among the most relatively abundant taxa (Fig 3B and 3C; LDA score ≥ 3.5). In terms of phyla, *Proteobacteria* and *Bacteroidetes* were more relatively abundant at day 0 of the experiment. Of the 15 most relatively abundant genera, only *Bacillus*, *Truepera*, and *Ureibacillus* were not enriched at one specific sampling time. At the genus level, *Arthrobacter*, *Pseudomonas* and *Psychrobacter* were found to be enriched at day 0. All three genera have been reported to be sensitive to thermophilic temperatures during composting, thus explaining their relative decline over time [44,45].

Taxa associated with either the middle or top sampling depth were also identified using LEfSe on day 14 and 99 samples. There were 12 phyla and 30 different genera that were enriched at one depth or the other, the majority of which were in the top depth (S1A and S1B Fig). The *Firmicutes* phylum was enriched in samples taken from the middle depth as were members of the *Euryarchaeota*. Meanwhile, *Proteobacteria* and *Bacteroidetes* were associated with the top sampling depth. This observation is in agreement with Meada et al. [20] who observed that 16S rRNA gene bands classified as *Bacteroidetes* and *Proteobacteria* were more dominant in the surface of composted cattle manure, and members of the *Firmicutes* at lower depths. Using 16S rRNA clone libraries, Tian et al. [44] also recorded a decrease in *Bacteroidetes* and *Proteobacteria*, and an increase in *Firmicutes*, after 12 days of composting dairy manure with rice chaff, relative to day 0. The middle depth had a higher temperature and prior to turning, oxygen levels would also be expected to be lower. This would explain why *Caldicoprobacter* and *Tepidimicrobium*, two genera of thermophilic, anaerobic bacteria, were most enriched at the middle depth [46].

Weighted UniFrac distances were also used to determine the effect of sampling time and sampling depth on the structure of the archaeal and bacterial microbiota. Samples taken at each time point clustered together, independent of compost mixture type (Fig 2B; P < 0.001; R-value = 0.41). Samples from days 14 and 99 also clustered together by sampling depth, although not as strongly (Fig 2C; P = 0.003; R-value = 0.22).

The finding that archaeal and bacterial diversity and richness was significantly reduced at day 99 is in accordance with a decrease in volatile fatty acid levels and water-extractable ions reported earlier for these samples at day 99 [18]. Bacterial diversity was also reported to be reduced in composted horse and cattle manure after 104 days [47] and in sewage sludge and cattle manure that was composted over 100 days [38]. Temperature represents the biggest factor behind the changes in the microbiota observed among sampling times and between sampling depths. The temperature within the compost bins at day 0 was approximately 35°C but at the middle depth of 90 cm, it increased to greater than 55°C within 5 days [18]. This rapid shift in temperature favours the thermophilic microbes initially present and that can readily adapt to the changing environmental conditions. Indeed, all of the enriched genera with the highest LDA scores at day 14 or 99 were present in the initial microbial population at day 0, and became more relatively abundant once the temperature increased. While archaeal and bacterial richness and diversity decreased from day 14 to 99, there was no significant change from day 0 to 14. In addition, there was a greater shift in the structure of the microbiota from day 14 to 99. This is likely a result of the longer period of time elapsed between the 2^nd^ and 3^rd^ sampling times, and thus a longer period of exposure to higher temperatures. It may also indicate that nutrients required for growth have started to become depleted by day 99, as by this time, total C was significantly reduced [18].

### Fungal ITS sequencing results

A total of 581,743 quality-filtered and chimera-checked ITS sequences were obtained having an average length of 276 bp. Only six different fungal phyla were identified in the compost samples among a total of 1076 unique OTUs. The large majority of these OTUs were low in abundance as the 10 most relatively abundant OTUs contained 62.3% of the ITS sequences (data not shown). Although all ITS sequences were identified as fungal in origin, overall greater than 55% of the sequences could not be assigned to a particular phylum. Of the ITS sequences that could be classified at the phylum level, the majority belonged to *Ascomycota*, with *Basidiomycota* and *Zygomycota* the only other phyla found in all compost samples. This is in agreement with previous studies where the dominance of the *Ascomycota* phylum was also noted in the fungal microbiota of composted manure [48,49].

A particular genus could also not be assigned to 70.2% of the ITS sequences. Despite this limitation, 120 fungal genera were identified across all compost samples with *Kernia* being the most relatively abundant genus at 12.2% of ITS sequences (Table 2). The OTUs classified as *Kernia* also had a species-level designation, with *Kernia pachypleura* being the sole species. Only four other genera comprised more than 1% of the total ITS sequences across all samples: *Remersonia* (*R*. *thermophila*), *Acremonium*, *Talaromyces*, and *Peziza*. Overall, 108 to 297 fungal OTUs were found in each compost sample (Table 3). A number of the other more relatively abundant genera, including *Thermomyces*, *Talaromyces*, *Acremonium*, *Fusarium*, *Microascus*, and *Aspergillus*, have also been frequently identified in earlier reports of the fungal microbiota in composts [43,48,49]. While *Aspergillus fumigatus* is a potentially pathogenic species of *Aspergillus* [50], this species was identified in only three samples and at very low abundance (< 0.05%; data not shown).

**Table 2:** The 15 most relatively abundant fungal genera detected throughout the composting of cattle manure^a^.

Phylum	Family	Genus	Species	% Total		
				Day 0	Day 14	Day 99
*Ascomycota*	*Microascaceae*	*Kernia*	*pachypleura*	26.88	6.62	2.96
*Ascomycota*	*Incertae sedis*	*Remersonia*	*thermophila*	2.88	11.23	0.81
*Ascomycota*	*Incertae sedis*	*Acremonium*	multiple	1.56	2.81	1.31
*Ascomycota*	*Trichocomaceae*	*Talaromyces*	*thermophilus*	0.14	0.08	3.90
*Ascomycota*	*Pezizaceae*	*Peziza*	*campestris*, *vesiculosa*	0.25	0.02	3.07
*Zygomycota*	*Mortierellaceae*	*Mortierella*	multiple	0.13	1.06	1.17
*Ascomycota*	*Incertae sedis*	*Thermomyces*	*lanuginosus*	0.06	0.41	1.88
*Ascomycota*	*Microascaceae*	*Microascus*	multiple	0.21	0.49	1.63
*Ascomycota*	*Pleosporaceae*	*Lewia*	*infectoria*	1.88	0.22	0.14
*Ascomycota*	*Trichocomacea*	*Aspergillus*	multiple	0.26	0.77	0.68
*Ascomycota*	*Lasiosphaeriaceae*	*Cladorrhinum*	*phialophoroides*	0.89	0.70	0.10
*Ascomycota*	*Helotiaceae*	*Rhizoscyphus*	*ericae*	0.88	0.33	0.05
*Basidiomycota*	*Filobasidiaceae*	*Cryptococcus*	multiple	0.28	0.68	0.18
*Basidiomycota*	*Incertae sedis*	*Myriococcum*	*thermophilum*	0.05	0.11	0.59

^**a**^ Fungal sequences were classified using the UNITE database. Species names are included for genera with two or less species detected. Percentage values are represented across all compost mixtures types.

**Table 3:** Fungal ITS sequence richness and diversity of composted cattle manure according to time and sampling depth.

Treatment^a^	Time	Depth^b^	Number of OTUs	Shannon	Chao1
CON	0	Top	142 ± 30b	2.28 ± 0.67	272 ± 90
CON C&D	0	Top	250 ± 57a	3.46 ± 0.88	425 ± 103
CON	14	Middle	237 ± 17ab	3.54 ± 0.16	379 ± 36
CON C&D	14	Middle	209 ± 34ab	3.06 ± 0.49	338 ± 50
CON	99	Middle	182 ± 40ab	2.79 ± 0.7	329 ± 58
CON C&D	99	Middle	166 ± 10ab	3.31 ± 0.23	283 ± 28

^a^ CON, manure from cattle on a control diet of barley; DDG, manure from cattle fed dried distillers grains and solubles; CON C&D, manure from the control diet cattle amended with construction and demolition waste. Mean ± standard deviation (n = 3) is shown for compost. Means with different lowercase letters are significantly different from one another (P<0.05).

^b^ Samples were taken at the top (7 cm) and middle (90 cm) of each compost bin.

The 50 most relatively abundant OTUs that were unclassified at the phylum-level were further analyzed using BLASTn and the representative sequence for each OTU (S4 Table). These OTUs comprised 50.5% of the total ITS sequences overall and among these sequences, *Kernia* was also identified as being the most relatively abundant (17.2% total relative abundance). *Kernia nitida* was the only species identified among the *Kernia* OTUs. An OTU with an overall relative abundance of 10.4% was identified as an uncultured *Pseudallescheria* spp., and two other OTUs with a relative abundance of 5.5% and 5.3% were classified as *Orpinomyces* spp. and *Hormographiella aspergillata*, respectively. Similar to OTUs classified within QIIME using the UNITE database, the overall relative abundance of the *Ascomycota* phylum was 39.4% and *Basidiomycota* 4.3% among these previously unclassified OTUs.

The fungal richness was nearly 10-fold less than the bacterial richness and fungal diversity was lower as well. This is expected given that feces from cattle contain significantly fewer fungi and fungi in general are also largely mesophilic and therefore unable to proliferate at temperatures higher than 55°C [51,52]. This has also been observed in other types of composting as well [49,53].

### Effect of compost mixture type and sampling time on the fungal microbiota

Fungal diversity and richness was not altered by either compost mixture type or sampling time (Table 3). The number of fungal OTUs in the CON compost was actually lower than the CON C&D compost at the start of the experiment (P < 0.05) but these two compost types did not differ at days 14 and 99. Using LEfSe, 15 different genera were enriched at one of the three sampling times (S2 Fig; LDA score greater than 3.0). Of these genera, *Kernia* was most notably enriched at day 0 (mesophilic phase) and *Remersonia* (*R*. *thermophila*) at day 14 (thermophilic phase). There were no genera that were differentially abundant between the CON and CON C&D samples at days 14 and 99 (LDA score less than 3.0). *Kernia* spp. are coprophilous and have been isolated from the feces of a number of different animals, including cattle [54], and have previously been reported to be among the more relatively abundant fungal genera in the composting of food and garden waste [48].

**Fig 4:**
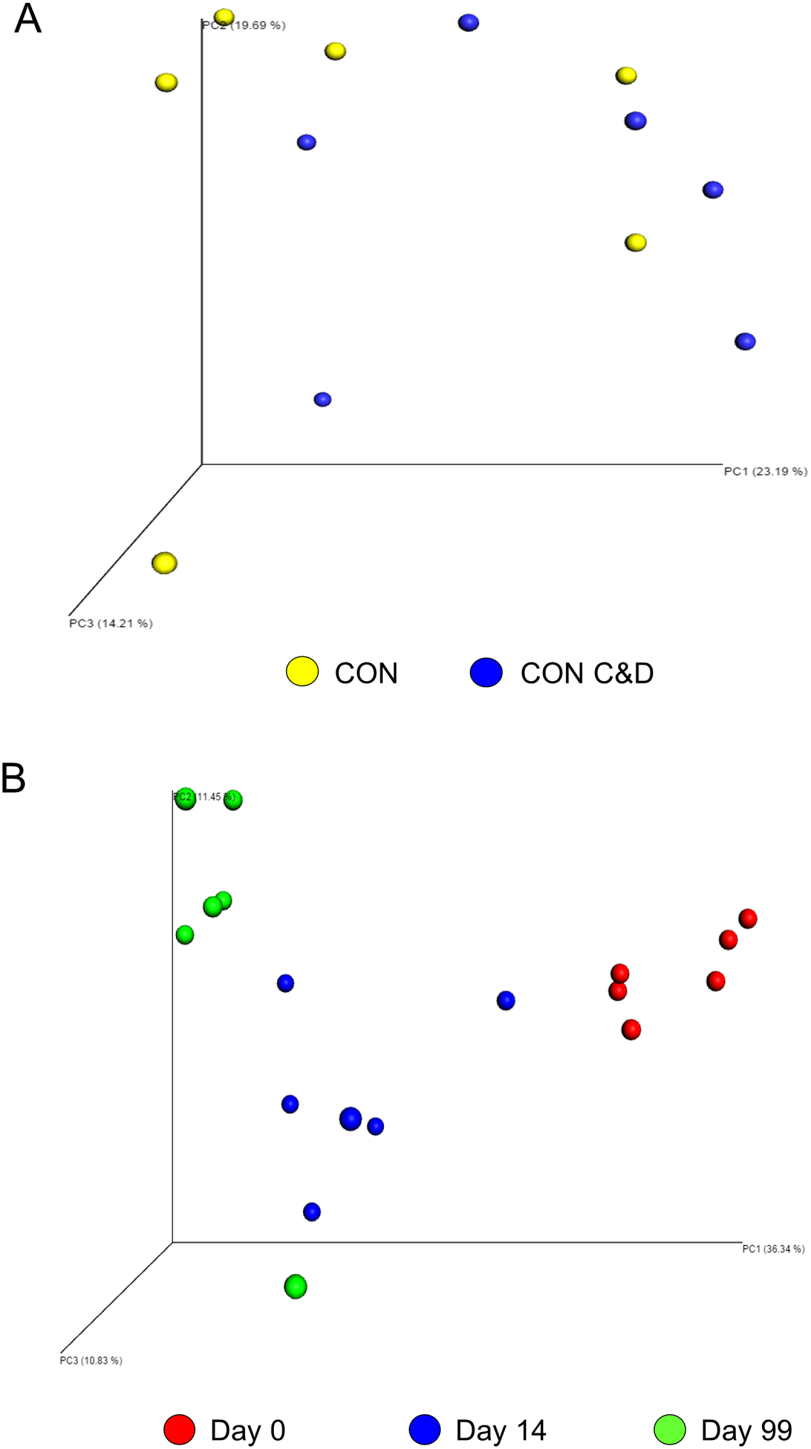
Principle coordinate analysis (PCoA) plots of the Bray-Curtis distances for the fungal microbiota for A) both compost mixture types (day 0 excluded) and B) each sampling time. CON = composted manure from cattle on a control diet of barley and CON C&D = composted manure from the control diet cattle amended with construction and demolition waste.

As the species name suggests, *R*. *thermophila* is a thermophilic fungus with an optimal growth temperature of 45°C [55], which likely explains the increase in relative abundance from day 0 to 14. *Microascus* (multiple species), *Talaromyces thermophilus*, and *Thermomyces lanuginosus*, were also found to be more relatively abundant at day 99. *T*. *thermophilus* and *T*. *lanuginosus* are both thermophiles with optimal growth temperatures similar to the ones recorded within the compost mixtures at day 99 [18,55]. The structure of the fungal community was assessed using the non-phylogenetic Bray-Curtis distance metric and plotted using PCoA (Fig 4). As with the archaeal and bacterial microbiota, the fungal microbiota structure was most affected by sampling time (Fig 4B; R-value = 0.69; P < 0.001). The addition of construction and demolition waste to the CON compost also did not alter the fungal community as the two compost mixture types did not cluster separately at days 14 or 99 (Fig 4A; P > 0.05). There were 11 OTUs that formed the core fungal microbiota of the composting process, as these OTUs were found in both sample types and all three sampling times (data not shown).

### Effect of compost mixture type, sampling time, and sampling depth on antimicrobial resistant determinants

Antimicrobial usage in agriculture remains a contentious issue due to its role in the development and dissemination of antimicrobial resistance determinants and resistant bacteria [56]. Agricultural soils that are amended with raw livestock manure are of particular concern as the concentrations of antimicrobial resistance determinants and resistant bacteria may be exceptionally high if applied directly without treatment [56]. In addition, resistant bacteria and resistance determinants found in livestock feces can potentially increase in concentration if left untreated after excretion [8,37]. Composting of manure prior to application has been shown to be an effective method to reduce antimicrobial resistance determinants in cattle manure, compared to stockpiling [11]. Therefore it is important to study whether changes in the initial composition of compost affects the ability to reduce antimicrobial resistance. To assess the effect that the composting process and the addition of C&D waste has on antimicrobial resistance in feedlot cattle manure, the concentrations of 12 antimicrobial resistance determinants were determined using qPCR.

**Fig 5:**
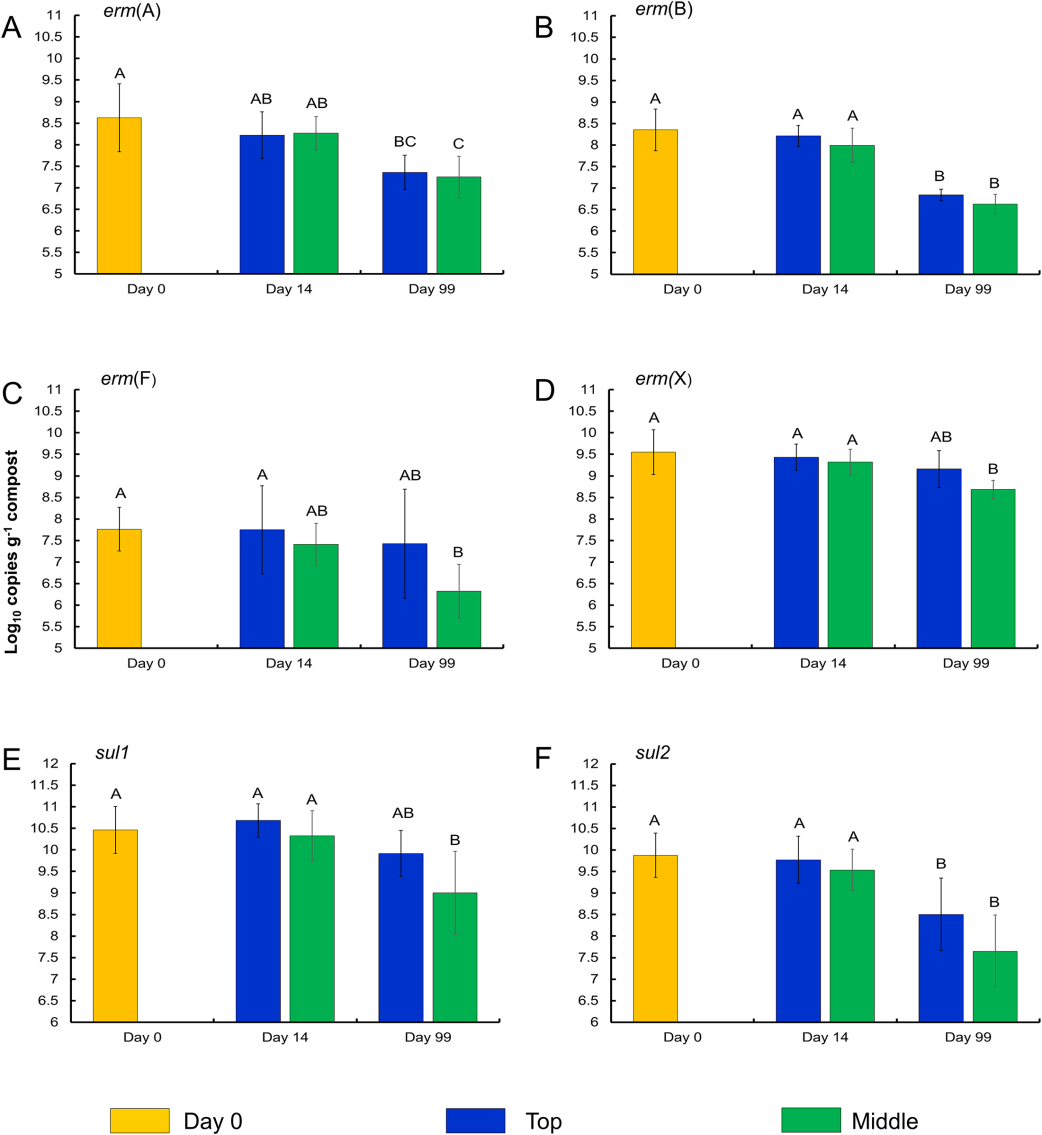
Concentrations of A) *erm*(A), B) *erm*(B), C) *erm*(F), D) *erm*(X), E) *sul1*, and F) *sul2*, by sampling time and depth. Error bars represent ± standard deviation of the mean (n = 9). All compost mixture types were combined for analysis as concentrations of each resistance determinants did not differ by compost mixture type at any one sampling time or depth (P > 0.05).

**Fig 6:**
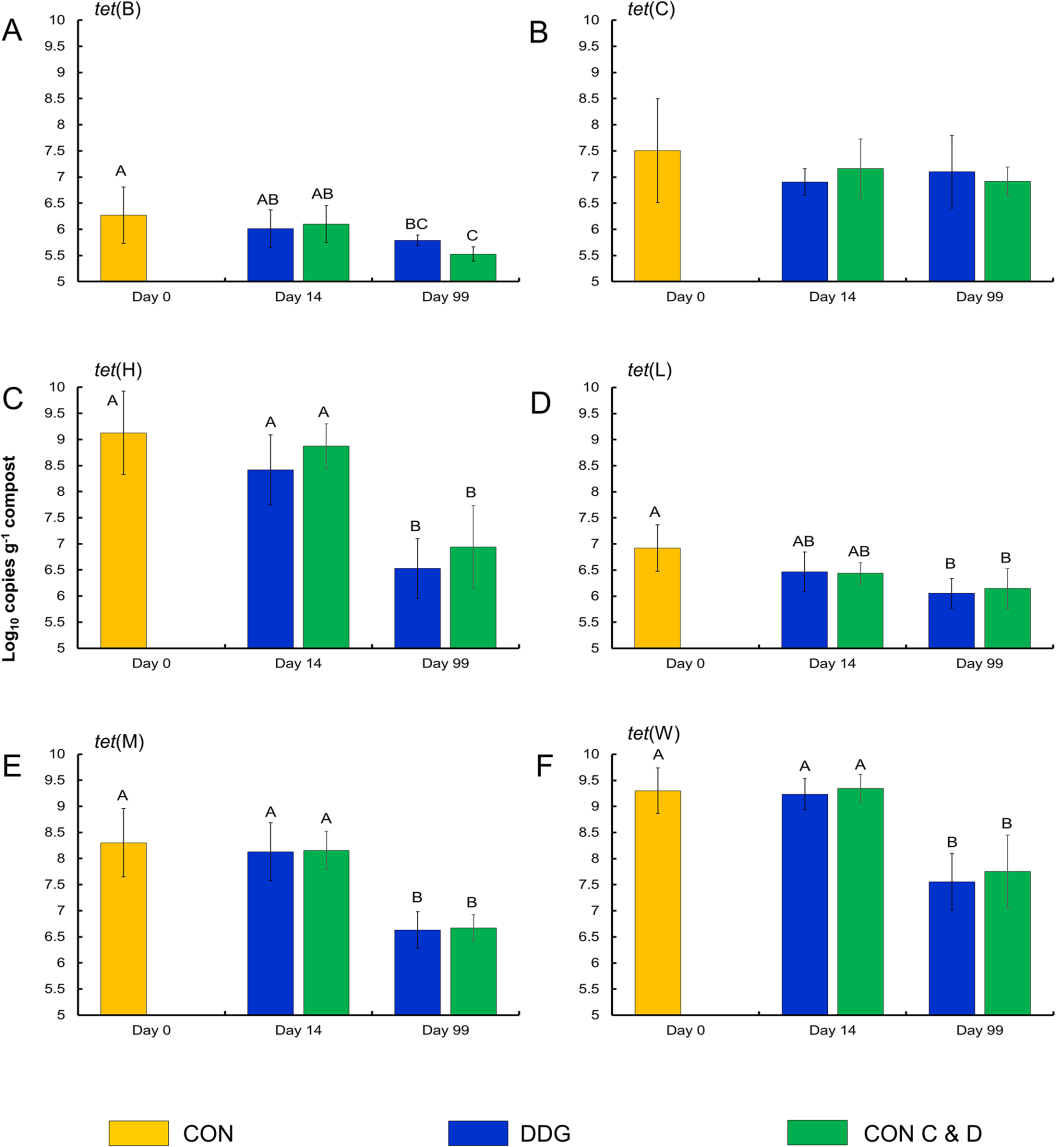
Concentrations of A) *tet*(B), B) *tet*(C), C) *tet*(H), D) *tet*(L), E) *tet*(M), and F) *tet*(W), by sampling time and depth. Error bars represent ± standard deviation of the mean (n = 9). All compost mixture types were combined for analysis as concentrations of each resistance determinants did not differ by compost mixture type at any one sampling time or depth (P > 0.05).

When comparing the concentrations (copies g^-1^ compost dry weight) of each resistance determinant, only time and depth had a significant effect (P < 0.05; Figs 5 and 6). All resistance determinants decreased in concentration over time (P < 0.05; Table 4), with the exception of *tet*(C) and *erm*(F) which both had similar concentrations at day 0 and day 99. Four of the resistance determinants were found at higher concentrations at the top (7 cm) of the compost pile compared with the middle depth (90 cm), while the concentration of *tet*(H) was actually higher at the middle depth (S5 Table). When comparing reductions in the concentration of each resistance determinant from day 0 to day 99, three resistance determinants (*erm*(A), *tet*(B), and *tet*(C)) were significantly reduced in the control compost compared to the DDG and CON C&W composts (Table 4). Interestingly, the *tet*(C) concentration actually increased in the DDG compost over the course of the study, although the reason for this finding is not clear as *tet*(C) decreased by 1.94 log_10_ copies g^-1^ compost dry weight in the CON compost. Numerically, concentrations of *tet*(H), *tet*(M), and *sul*2 were observed to have the largest reductions. The resistance determinants with the highest concentrations at day 0 among all compost samples were as follows in descending order: *sul1*, *sul2*, *erm*(X), *tet*(W), *tet*(H), *erm*(A), *erm*(B), *tet*(M), *erm*(F), *tet*(C), *tet*(L), and *tet*(B) (Figs 5 and 6).

**Table 4:** Log_10_ reductions in the concentration (copies g^-1^ compost dry weight) of 12 different antimicrobial resistance determinants from day 0 to day 99 in composted manure from cattle^a^.

Resistance determinant	CON	DDG	CON C&D
*erm*(A)	-1.51 ± 0.37	-1.28 ± 0.51	-1.17 ± 1.28
*erm*(B)	-1.71 ± 0.52	-1.66 ± 0.31	-1.50 ± 0.57
*erm*(F)	-0.92 ± 1.38	-0.28 ± 1.01	-1.46 ± 0.87
*erm*(X)	-1.00 ± 0.42	-0.46 ± 0.4	-0.42 ± 0.64
***tet*(B)**	**-1.15 ± 0.23a**	**-0.45 ± 0.26b**	**-0.23 ± 0.62b**
***tet*(C)**	**-1.94 ± 0.38b**	**0.45 ± 0.47a**	**0.01 ± 1.09a**
*tet*(H)	-2.79 ± 0.62	-1.86 ± 0.45	-2.53 ± 1.46
*tet*(L)	-0.97 ± 0.38	-0.61 ± 0.29	-0.88 ± 0.82
***tet*(M)**	**-2.6 ± 0.58b**	**-1.63 ± 0.38a**	**-1.37 ± 0.60a**
*tet*(W)	-1.55 ± 0.41	-1.52 ± 0.30	-1.87 ± 0.96
*sul1*	-0.66 ± 0.74	-0.88 ± 0.43	-1.47 ± 1.01
*sul2*	-1.37 ± 1.00	-1.62 ± 0.47	-2.41 ± 0.81

^**a**^ CON, manure from cattle on a control diet of barley; DDG, manure from cattle fed dried distillers grains and solubles; CON C&D, manure from the control diet cattle amended with construction and demolition waste. Mean ± standard deviation (n = 6) with top and middle depths combined for analysis. Means with different lowercase letters and in bold in rows are significantly different from one another (P < 0.05).

As with the reduction in archaeal and bacterial diversity, the extended exposure to temperatures higher than 55°C is most likely responsible for the significant decrease in concentration observed for 10 of the 12 antimicrobial resistance determinants over the course of the study. This is further evidenced by the fact that at the middle depth, where the temperature was higher, significantly lower concentrations were observed for four antimicrobial resistance determinants. The concentrations observed for all the resistance determinants in the compost mixtures at day 0 were similar to those reported previously in feces from cattle at this same feedlot [37].

Pearson correlation analysis was used to assess the association between the concentration of each resistance determinant and the proportion of the 20 most relatively abundant bacterial genera (S6 Table). Six genera showed a significant correlation between relative abundance and the concentration of at least one resistance determinant. While four of these genera had positive correlations, *Psychrobacter* and *Proteiniphilum* were negatively associated with *sul1* and/or *sul2*. This is an interesting finding given that *Psychrobacter* isolates from soil and swine slurry have been shown to carry both *sul1* and *sul2* [57]. *Corynebacterium*, *Anoxybacillus*, *Tepidimicrobium*, and *Dietzia*, were moderately (r = 0.3 to 0.5) correlated with the concentration of four or more resistance determinants, namely *tet*(B), *tet*(H), *tet*(M), *tet*(W), *erm*(A), and *erm*(B). Only *tet*(C) was not correlated with the relative abundance at least one bacterial genus. It is unclear whether these associations represent a higher prevalence of these genes among the four genera; however, *Corynebacterium* and *Dietzia* isolated from swine manure pits and associated soils have been reported to be reservoirs of tetracycline resistance determinants, including *tet*(B) [58]. In fact, five *tet* and four *erm* genes have been identified within the *Corynebacterium* genus, including *tet*(M), *tet*(W), *erm*(B), and *erm*(X) (http://faculty.washington.edu/marilynr/).

## Conclusions

Overall, neither the source of the manure nor the addition of C&D waste, was a significant factor in determining the structure of the compost microbiota. Instead, the compost microbiota changed over time and by sampling depth. The concentrations of antimicrobial resistance determinants also changed in a similar way during the composting process, with concentrations of all but two resistance determinants lowered by day 99. Importantly, as the addition of C&D waste did not alter the compost microbiota, its inclusion in composted manure offers a safe, viable option for diverting C&D waste from landfills.

## Supporting Information

S1 Fig Differentially abundant bacterial and archaeal A) phyla and B) genera, at one of the two sampling depths as assessed using linear discriminant analysis (LDA) with effect size measurements (LEfSe). (TIF)

S2 Fig Differentially abundant fungal genera across the two compost mixture types (CON, CON C&D) as assessed using linear discriminant analysis (LDA) with effect size measurements (LEfSe). (TIF)

S1 Table Primers and annealing temperatures used for qPCR assays. (DOCX)

S2 Table Percent relative abundance of phyla detected in compost samples by mixture type, sampling depth, and time. (XLSX)

S3 Table Bacterial operational taxonomic units (OTUs) found in all samples at all sampling times, depths, and compost mixture types. (XLSX)

S4 Table BLASTn results for the 50 most relatively abundant OTUs that were unclassified using the UNITE database. (XLSX)

S5 Table Concentrations (copies g^-1^ compost dry weight) of each resistance determinant at the top (0 cm) and middle (90 cm) sampling depths of composted manure from cattle. (DOCX)

S6 Table Pearson’s correlation coefficients between the proportion of the 20 most relatively abundant bacterial genera and the number of copies of 12 different resistance determinants (log_10_ copies g^-1^ compost dry weight). (DOCX)
