# Supplementary figures and images for: Effect of Co-Composting Cattle Manure with Construction and Demolition Waste on the Archaeal, Bacterial, and Fungal Microbiota, and on Antimicrobial Resistance Determinants
Source: PLoS One. 2016 Jun 14;11(6):e0157539. doi: 10.1371/journal.pone.0157539 (PMC4907429; doi:10.1371/journal.pone.0157539)

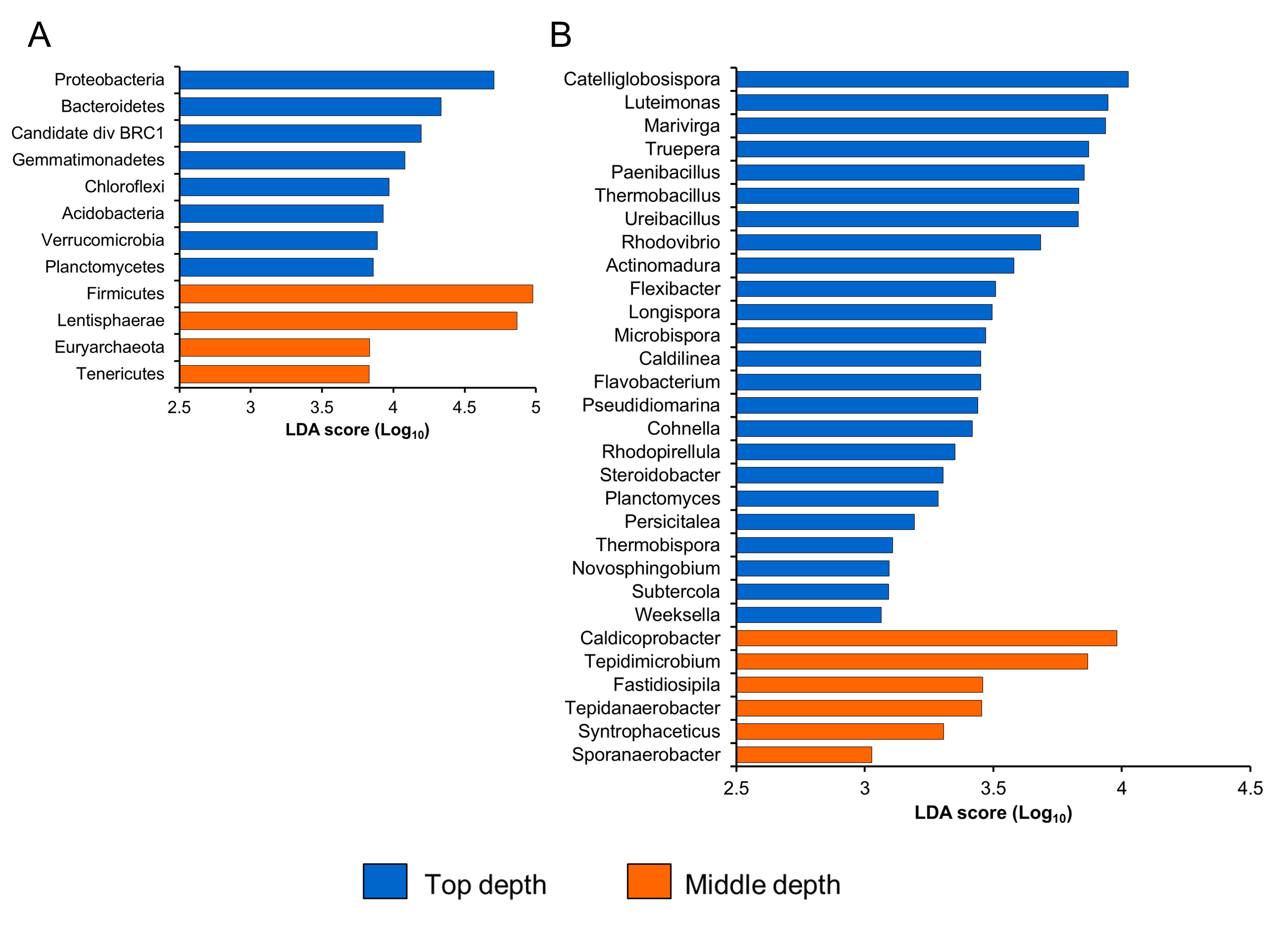

Supplement: S1 Fig — (TIF) [file pone.0157539.s001.tif]

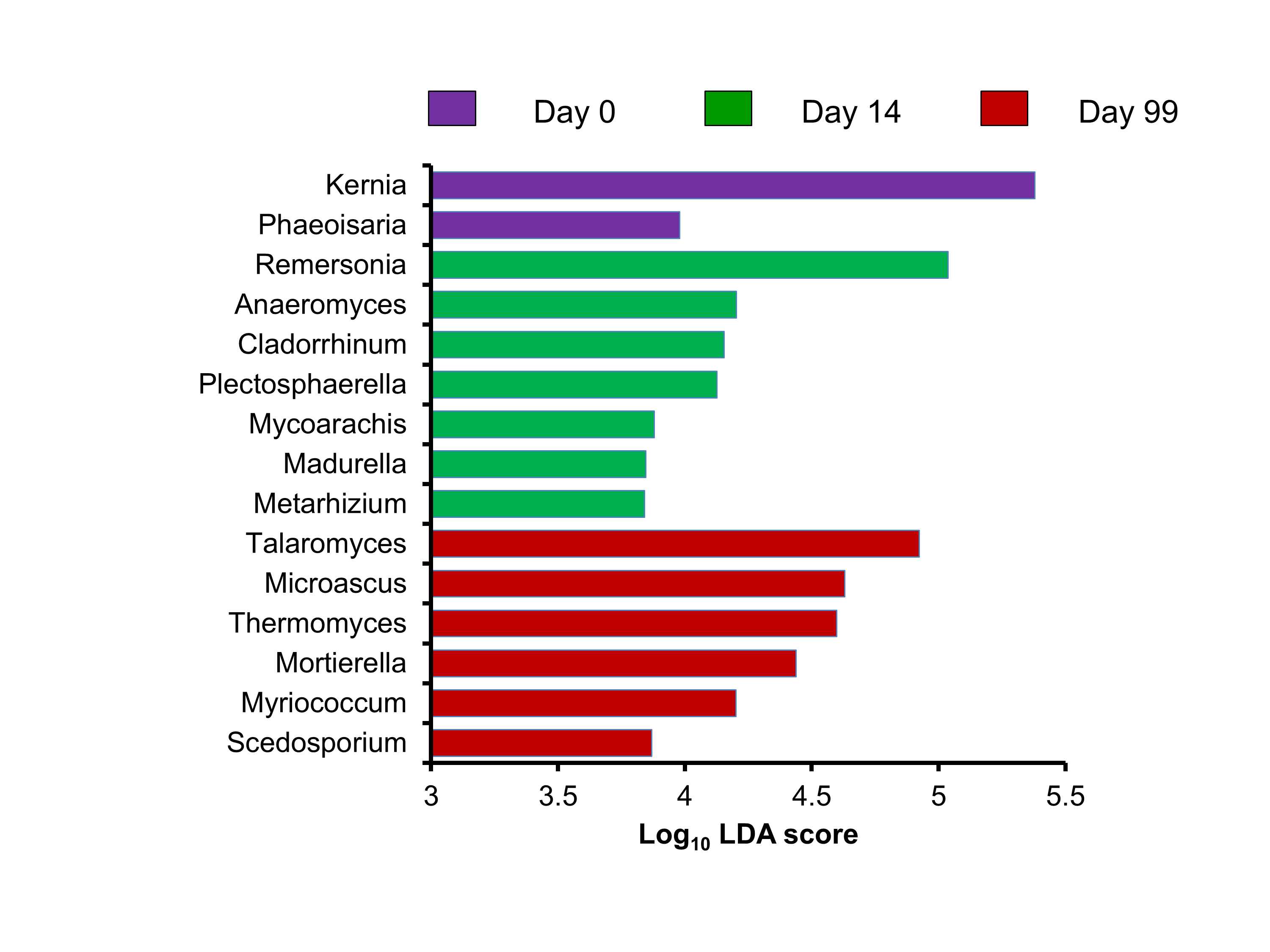

Supplement: S2 Fig — (TIF) [file pone.0157539.s002.tif]
