# Supplementary material for: Effect of Co-Composting Cattle Manure with Construction and Demolition Waste on the Archaeal, Bacterial, and Fungal Microbiota, and on Antimicrobial Resistance Determinants
Source: PLoS One. 2016 Jun 14;11(6):e0157539. doi: 10.1371/journal.pone.0157539 (PMC4907429; doi:10.1371/journal.pone.0157539)
Supplement: S1 Table — (DOCX) [file pone.0157539.s003.docx]

| Target | Sequence (5’to 3’) | Annealing temperature (°C) | Amplicon size (bp) | Reference |  |
| --- | --- | --- | --- | --- | --- |
| *tet*(B) | ACACTCAGTATTCCAAGCCTTTG | 60 | 205 | [1] |  |
|  | GATAGACATCACTCCCTGTAATGC |  |  |  |  |
| *tet*(C) | CTTGAGAGCCTTCAACCCAG | 55 | 418 | [2] |  |
| *tet*(H) | ATGGTCGTCATCTACCTGCC  CAGTGAAAATTCACTGGCAAC  ATCCAAAGTGTGGTTGAGAAT | 56 | 185 | [3] |  |
| *tet*(L) | TCGTTAGCGTGCTGTCATTC | 55 | 267 | [2] |  |
|  | GTATCCCACCAATGTAGCCG |  |  |  |  |
| *tet*(M) | GTGGACAAAGGTACAACGAG | 55 | 406 | [2] |  |
|  | CGGTAAAGTTCGTCACACAC |  |  |  |  |
| *tet*(W) | GAGAGCCTGCTATATGCCAGC | 64 | 168 | [4] |  |
|  | GGGCGTATCCACAATGTTAAC |  |  |  |  |
| *erm*(A) | GAAATYGGRTCAGGAAAAGG | 54 | 332 | [5] |  |
|  | AAYAGYAAACCYAAAGCTC |  |  |  |  |
| *erm*(B) | GATACCGTTTACGAAATTGG | 58 | 364 | [5] |  |
|  | GAATCGAGACTTGAGTGTGC |  |  |  |  |
| *erm*(F) | CGACACAGCTTTGGTTGAAC | 56 | 309 | [5] |  |
|  | GGACCTACCTCATAGACAAG |  |  |  |  |
| *erm*(X) | GAGATCGGRCCAGGAAGC | 58 | 488 | [5] |  |
|  | GTGTGCACCATCGCCTGA |  |  |  |  |
| *sul*(1) | CGCACCGGAAACATCGCTGCAC | 56 | 163 | [6] |  |
|  | TGAAGTTCCGCCGCAAGGCTCG |  |  |  |  |
| *sul*(2) | TCCGGTGGAGGCCGGTATCTGG | 61 | 191 | [6] |  |
|  | CGGGAATGCCATCTGCCTTGAG |  |  |  |  |

**S1 Table**. **Primers and annealing temperatures used for qPCR assays.**

1. Peak N, Knapp CW, Yang RK, Hanfelt MM, Smith MS, Aga DS, et al. Abundance of six tetracycline resistance genes in wastewater lagoons at cattle feedlots with different antibiotic use strategies. Environ Microbiol. 2007;9(1):143-51.

2. Ng LK, Martin I, Alfa M, Mulvey M. Multiplex PCR for the detection of tetracycline resistant genes. Mol Cell Probes. 2001;15(4):209-15. doi: 10.1006/mcpr.2001.0363. PubMed PMID: 11513555.

3. Aminov RI, Chee-Sanford JC, Garrigues N, Teferedegne B, Krapac IJ, White BA, et al. Development, validation, and application of PCR primers for detection of tetracycline efflux genes of gram-negative bacteria. Appl Environ Microbiol. 2002;68(4):1786-93. PubMed PMID: 11916697; PubMed Central PMCID: PMC123860.

4. Aminov RI, Garrigues-Jeanjean N, Mackie RI. Molecular ecology of tetracycline resistance: development and validation of primers for detection of tetracycline resistance genes encoding ribosomal protection proteins. Appl Environ Microbiol. 2001;67(1):22-32. doi: 10.1128/AEM.67.1.22-32.2001. PubMed PMID: 11133424; PubMed Central PMCID: PMC92507.

5. Chen J, Yu Z, Michel FC, Jr., Wittum T, Morrison M. Development and application of real-time PCR assays for quantification of erm genes conferring resistance to macrolides-lincosamides-streptogramin B in livestock manure and manure management systems. Appl Environ Microbiol. 2007;73(14):4407-16. doi: 10.1128/AEM.02799-06. PubMed PMID: 17496134; PubMed Central PMCID: PMC1932836.

6. Negreanu Y, Pasternak Z, Jurkevitch E, Cytryn E. Impact of treated wastewater irrigation on antibiotic resistance in agricultural soils. Environ Sci Technol. 2012;46(9):4800-8. doi: 10.1021/es204665b. PubMed PMID: 22494147.
