# Supplementary material for: Effect of Co-Composting Cattle Manure with Construction and Demolition Waste on the Archaeal, Bacterial, and Fungal Microbiota, and on Antimicrobial Resistance Determinants
Source: PLoS One. 2016 Jun 14;11(6):e0157539. doi: 10.1371/journal.pone.0157539 (PMC4907429; doi:10.1371/journal.pone.0157539)
Supplement: S5 Table — (DOCX) [file pone.0157539.s007.docx]

| **Resistance determinant** | **Top** | **Middle** | **P-value** |
| --- | --- | --- | --- |
| *erm*(A) | 7.79 ± 0.64 | 7.76 ± 0.67 | 0.434 |
| *erm*(B) | 7.53 ± 0.73 | 7.31 ± 0.77 | 0.070 |
| ***erm*(F)** | **7.59 ± 1.13** | **6.87 ± 0.78** | **0.040** |
| ***erm*(X)** | **9.30 ± 0.39** | **9.00 ± 0.41** | **0.033** |
| *tet*(B) | 5.90 ± 0.28 | 5.81 ± 0.39 | 0.310 |
| *tet*(C) | 7.00 ± 0.52 | 7.04 ± 0.45 | 0.782 |
| ***tet*(H)** | **7.47 ± 1.14** | **7.91 ± 1.17** | **0.049** |
| *tet*(L) | 6.26 ± 0.39 | 6.29 ± 0.33 | 0.772 |
| *tet*(M) | 7.34 ± 0.94 | 7.24 ± 1.06 | 0.519 |
| *tet*(W) | 8.40 ± 0.96 | 8.55 ± 0.97 | 0.317 |
| ***sul1*** | **10.30 ± 0.60** | **9.67 ± 1.03** | **0.014** |
| ***sul2*** | **9.14 ± 0.95** | **8.59 ± 1.18** | **0.039** |

**S5 Table**. **Concentrations (copies g^-1^ compost dry weight) of each resistance determinant at the top (0 cm) and middle (90 cm) sampling depths of composted manure from cattle.** Mean ± standard deviation (n=18) with all compost treatments and days 14 and 99 combined for analysis. Means in rows that are in bold are significantly different from one another (P < 0.05).
