# Supplementary material for: Effect of Co-Composting Cattle Manure with Construction and Demolition Waste on the Archaeal, Bacterial, and Fungal Microbiota, and on Antimicrobial Resistance Determinants
Source: PLoS One. 2016 Jun 14;11(6):e0157539. doi: 10.1371/journal.pone.0157539 (PMC4907429; doi:10.1371/journal.pone.0157539)
Supplement: S6 Table — (DOCX) [file pone.0157539.s008.docx]

**S6 Table . Pearson’s correlation coefficients between the proportion of the 20 most relatively abundant bacterial genera and the number of copies of 12 different resistance determinants (log_10_ copies g^-1^ compost dry weight).**

| **Genus** | ***tet*(B)** | ***tet*(C)** | ***tet*(H)** | ***tet*(L)** | ***tet*(M)** | ***tet*(W)** | ***erm*(A)** | ***erm*(B)** | ***erm*(F)** | ***erm*(X)** | ***sul1*** | ***sul2*** |
| --- | --- | --- | --- | --- | --- | --- | --- | --- | --- | --- | --- | --- |
| *Bacillus* | -0.21 | -0.12 | 0.03 | 0.08 | -0.05 | 0.10 | -0.03 | -0.06 | -0.20 | -0.08 | -0.03 | -0.08 |
| *Halocella* | -0.11 | -0.09 | 0.07 | 0.00 | 0.00 | 0.09 | -0.03 | 0.05 | -0.11 | -0.10 | -0.02 | 0.03 |
| *Corynebacterium* | 0.23 | 0.09 | **0.34*** | 0.22 | **0.33*** | **0.33*** | **0.31*** | **0.42**** | 0.10 | **0.31*** | 0.23 | 0.25 |
| *Pseudomonas* | **0.33*** | 0.24 | 0.19 | 0.16 | 0.23 | 0.10 | 0.26 | 0.24 | 0.05 | 0.23 | 0.03 | 0.03 |
| *Anoxybacillus* | **0.38*** | 0.16 | **0.37*** | 0.29 | **0.40**** | **0.40**** | **0.33*** | **0.43**** | **0.37*** | **0.36*** | **0.33*** | **0.40**** |
| *Arthrobacter* | 0.08 | 0.06 | 0.04 | -0.07 | 0.07 | -0.03 | 0.03 | 0.11 | -0.13 | 0.09 | -0.04 | -0.10 |
| *Psychrobacter* | 0.00 | 0.13 | -0.23 | -0.27 | -0.18 | -0.33 | -0.19 | -0.17 | -0.26 | -0.12 | **-0.35*** | **-0.39**** |
| *Truepera* | 0.24 | 0.05 | 0.03 | 0.25 | 0.16 | 0.06 | 0.23 | 0.15 | 0.21 | 0.18 | 0.11 | 0.14 |
| *Caldicoprobacter* | -0.15 | -0.05 | 0.13 | -0.03 | 0.04 | 0.13 | -0.01 | 0.04 | -0.10 | -0.05 | 0.03 | 0.04 |
| *Thermobifida* | -0.20 | 0.05 | -0.15 | -0.10 | -0.16 | -0.15 | -0.13 | -0.24 | 0.03 | -0.06 | 0.00 | -0.06 |
| *Planifilum* | -0.14 | 0.10 | -0.10 | -0.05 | -0.11 | -0.10 | -0.06 | -0.21 | 0.05 | -0.03 | 0.00 | -0.04 |
| *Tepidimicrobium* | 0.24 | 0.11 | **0.45**** | **0.34*** | **0.30*** | **0.43**** | **0.39**** | **0.42**** | 0.13 | 0.23 | 0.26 | **0.34*** |
| *Ureibacillus* | 0.09 | 0.20 | 0.03 | 0.21 | 0.05 | 0.02 | 0.04 | 0.02 | -0.05 | -0.05 | -0.21 | -0.04 |
| *Haloplasma* | -0.16 | -0.12 | 0.08 | -0.03 | -0.02 | 0.09 | -0.06 | 0.02 | -0.16 | -0.18 | -0.06 | -0.01 |
| *Jeotgalicoccus* | 0.18 | 0.19 | 0.03 | -0.03 | 0.09 | -0.05 | 0.05 | 0.13 | -0.01 | 0.13 | -0.02 | -0.07 |
| *Thermobacillus* | -0.02 | -0.25 | -0.07 | 0.01 | -0.09 | -0.02 | -0.03 | 0.00 | 0.11 | -0.07 | 0.10 | 0.11 |
| *Dietzia* | **0.32*** | 0.18 | **0.33*** | 0.20 | 0.35 | 0.26 | **0.30*** | **0.39**** | 0.15 | 0.28 | 0.18 | 0.24 |
| *Proteiniphilum* | 0.04 | 0.15 | -0.19 | -0.20 | -0.16 | **-0.31*** | -0.13 | -0.11 | -0.22 | -0.05 | -0.26 | **-0.33*** |
| *Geobacillus* | -0.13 | 0.13 | -0.17 | -0.15 | -0.15 | -0.20 | -0.09 | -0.24 | 0.05 | 0.03 | -0.01 | -0.05 |
| *Caldalkalibacillus* | -0.19 | 0.04 | -0.05 | -0.07 | -0.11 | -0.03 | -0.14 | -0.18 | -0.09 | -0.12 | -0.06 | -0.08 |

* P < 0.05

** P < 0.01
